# Measurement of Core Body Temperature Using Graphene-Inked Infrared Thermopile Sensor

**DOI:** 10.3390/s18103315

**Published:** 2018-10-03

**Authors:** Jorge S. Chaglla E., Numan Celik, Wamadeva Balachandran

**Affiliations:** 1 Department of Electronics and Computer Engineering, Brunel University London, Kingston Lane, Uxbridge UB8 3PH, UK; jsanticabj@hotmail.com (J.S.C.E.); wamadeva.balachandran@brunel.ac.uk (W.B.); 2 Institute of Pharmaceutical Science, King’s College London, London SE1 9NH, UK

**Keywords:** core body temperature, graphene ink, infrared thermopile sensor, distant measurement, ear canal sensing

## Abstract

Continuous and reliable measurements of core body temperature (CBT) are vital for studies on human thermoregulation. Because tympanic membrane directly reflects the temperature of the carotid artery, it is an accurate and non-invasive method to record CBT. However, commercial tympanic thermometers lack portability and continuous measurements. In this study, graphene inks were utilized to increase the accuracy of the temperature measurements from the ear by coating graphene platelets on the lens of an infrared thermopile sensor. The proposed ear-based device was designed by investigating ear canal geometry and developed with 3D printing technology using the Computer-Aided Design (CAD) Software, SolidWorks 2016. It employs an Arduino Pro Mini and a Bluetooth module. The proposed system runs with a 3.7 V, 850 mAh rechargeable lithium-polymer battery that allows long-term, continuous monitoring. Raw data are continuously and wirelessly plotted on a mobile phone app. The test was performed on 10 subjects under resting and exercising in a total period of 25 min. Achieved results were compared with the commercially available Braun Thermoscan, Original Thermopile, and Cosinuss One ear thermometers. It is also comprehended that such system will be useful in personalized medicine as wearable in-ear device with wireless connectivity.

## 1. Introduction

Core body temperature (CBT) is one of the key vitals of human health for diagnosing and preventing serious climatic injuries, such as hypothermia, hyperthermia, and heat stroke, especially during exercise, sport activities and infectious diseases. Human body normally keeps CBT within a specific range between 36.1 °C and 37.2 °C, by the thermoregulatory centre in the hypothalamus [1]. The main aim of monitoring CBT is to capture any signs of systematic infection in the presence of fever or to discover whether it is too high above normal temperature. In healthcare environments, monitoring CBT is crucial for detecting deterioration of patient conditions [2]. Screening of febrile conditions has been used even in airports to prevent massive transmission of respiratory infections [3]. Therefore, it is essential to detect even minor changes in core body temperature. 

Due to relevance of CBT, several methods and devices have been developed to measure it noninvasively. Ear canal is a suitable place in the body to record CBT as a non-invasive method, because the tympanic membrane in the ear canal directly reflects the CBT [4,5]. Hence, earphone-type infrared thermometers are being used to record CBT continuously from the ear [6,7,8]. However, the proposed ear-based infrared sensors are not good enough to obtain CBT accurately due to positioning in the ear canal at the incorrect angle and also incapacity of electromechanical and thermal properties of the sensor material. Graphene (GN), which is a recently discovered material, has largely attracted the attention of the research community due to its outstanding properties; namely, the highest surface area, the best electrical and thermal conductivity and mechanical strength, and the highest electron mobility of any material [9,10].

This work, hence, aimed at proposing and validating a low cost, energy efficient, reliable measurement and a portable solution to continuously and wirelessly monitor CBT in an unobtrusive manner. Towards this goal, a novel sensor system was developed by coating graphene platelets onto the surface of the lens of an infrared thermopile sensor using a drop-casting technique to increase the accuracy of the temperature measurements. It is known that graphene is a highly conductive material and it has been demonstrated that the electromechanical characteristics of composite materials can be enhanced in such biomedical applications [11,12]. Furthermore, it has been determined that, with an absorbance of nearly 2.3%, graphene shows a strong optical absorption in the infrared range due to intraband transitions [13]. This outstanding optical property of single layer graphene might reduce the scatter of IR light and therefore increase the amount of IR energy collected by the thermopile of the sensor. 

The proposed ear-based device was formed by 3D printing technology and includes an Arduino Pro mini for signal processing, a Bluetooth module for wireless transmission of CBT data, and a rechargeable Lithium battery for power source. The study was carried out with 10 volunteers in two different phases of activity: (1) during 6 min of complete rest; and (2) after 10 min of exercise. The results obtained from the proposed graphene-coated earplug device were then compared with three different types of commercially available sensors in this work.

## 2. Materials and Methods

The CBT measurement was performed using a commercial infrared (IR) thermopile MLX90614-DCA (developed by Melexis, Ypres, Belgium) [14]. This small, non-contact thermometer is composed of a factory calibrated IR thermopile detector together with a signal processing unit, which includes 17-bit analogue-to-digital conversion and digital signal processing. It provides an accuracy suitable for medical applications of ±0.2 °C in the range from 36 °C to 38 °C.

**Figure 1:**
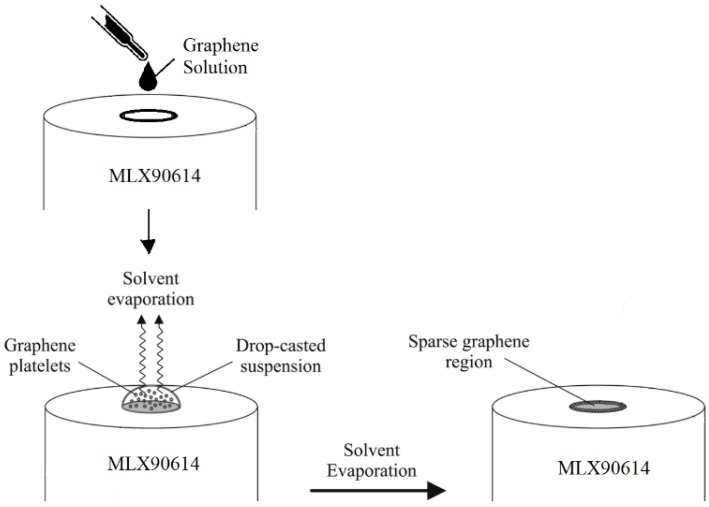
Coating process to obtain the graphene-inked thermopile. Adapted from [15].

To fabricate the graphene-inked sensor, a drop-casting method was used for coating graphene on top of the MLX90614-DCA thermopile as explained in Figure 1. A single channel pipette Finnpipette II was employed to drop-cast 3 μL of Aquagraph solution (obtained from 2D-TECH) over the silicon substrate of the lens of the thermopile. This solution contains graphene platelets in isopropyl alcohol (IPA) at a concentration of 2 mg/mL. Then, the sensor was left to dry for 24 h at ambient temperature to ensure the attachment of the graphene platelets to the lens of the thermopile.

The sensing device was developed to continuously measure CBT from the tympanic membrane and to monitor this vital sign on a smartphone. An Arduino Pro Mini board, which embeds an ATmega 328 microcontroller, collects the temperature measured from the MLX90614-DCA thermopile through the System Management Bus (SMBus) protocol and transmits it via Bluetooth to an Android app. Wireless connectivity is performed using a Bluetooth Mate Silver, which features an RN-42 Class 2 Bluetooth module, at a baud rate of 115,200 bits per second (bps). The CBT system is powered using a 3.7 V, 850 mAh rechargeable lithium-polymer battery that provides a continuous operation of the device for at least 4 h.

An ear hook-type enclosure was designed using CAD software and fabricated using 3D printing techniques. The ear hook holds the device attached to the auricle. The shape of the hook is based on the approximated curvature of the ear root, as measured by Lee et al. [16], as well as the geometrical dimensions of the auricle given by the British Standards Institution [17]. The designed parts were built from Accura Extreme resin using the ViperTM SLA^®^ system 3D printer, which uses stereolithography (STL) technology. Furthermore, the MLX90614-DCA thermopile was covered with a silicone cushion that, besides giving the user a comfortable fit, avoids the risk of cross-infection as a new cushion is used for each participant. 

Two experiments were executed to evaluate the performance of the graphene-inked sensor. The first one evaluated the system on participants at rest, whereas the second aimed to evaluate if the system can detect changes in CBT on participants performing physical activity. A total of 10 subjects, four males and six females, between 24 and 29 years old, were enrolled. All subjects were healthy, did not suffer from any disease and did not present febrile conditions. Participants were recruited on a voluntary basis, and they signed an informed consent for their participation in the trial. 

Under rest, CBT measurements from the right ear were taken with each participant seated and resting. The protocol involved the registration of temperature once every 60 s during 10 min with four different devices. An infrared tympanic thermometer (IRTT) ThermoScan^®^ 7 Age Precision^®^—IRT6520 (Braun GmbH, Kronberg, Germany) was used as the reference. Tympanic temperature readings were recorded with the designed CBT system using both the original MLX90614-DCA thermopile and the graphene-inked thermopile. 

For the test under physical activity, one 26-year-old female participant was enrolled. The experiment consisted of recording the CBT using the same four devices as in the previous test, while the participant was exercising on a cross trainer machine in an outdoor gym. The test was performed on a cloudy day at 21 °C ambient temperature. Measurements were recorded during 25 min. In this test, additional body temperature measurements given by a portable body sensor monitor Cosinuss One (Cosinuss GmbH, München, Germany) were also documented.

## 3. Results and Discussion

### 3.1. Sensor Characterization

**Figure 2:**
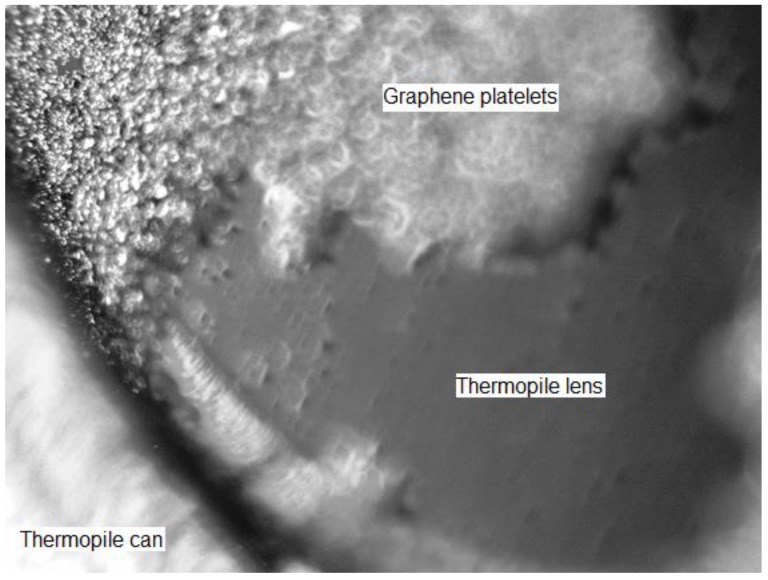
Amplified image of the graphene-inked MLX90614 thermopile.

Unfortunately, Raman spectra and Scanning Electron Micrographs could not be obtained as the electronic components inside the sensor would suffer damage causing the IR thermopile to malfunction. Therefore, the resultant coating of graphene was observed using an Olympus BH-2 optical microscope at 250× amplification. Figure 2 shows the edge between the lens and the metal can of the thermopile package. Graphene platelets are accumulated at some zones of the lens while other parts of the surface are uncovered. Overall, 70% of the lens surface was covered with graphene. Furthermore, there is a higher accumulation of graphene platelets at the edges of the lens. Therefore, the graphene coating obtained consists of various layers of graphene platelets not uniformly distributed around the lens of the IR thermopile.

### 3.2. Device Operation

**Figure 3:**
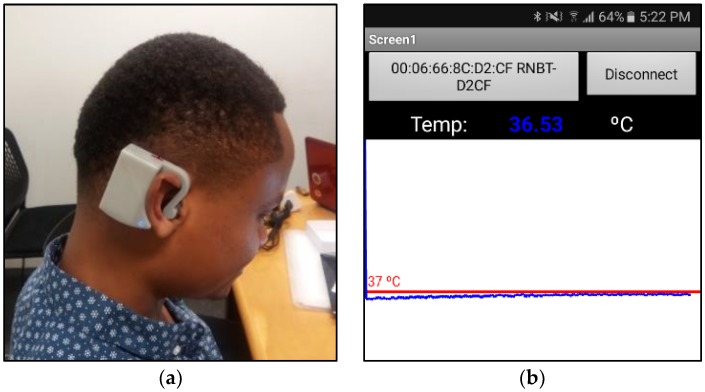
(**a**) Assembled device; and (**b**) monitoring of CBT on the smartphone.

Figure 3 shows the experimental CBT measurement device and the CBT results on the smartphone. The assembled device has a total weight of 44.8 gm. and dimensions of 58 mm × 58 mm × 21 mm. The ear hook adapts to the shape of the auricle holding and supporting the weight of the components. Moreover, all subjects considered it comfortable to wear during the 10 min that the experiment lasted.

### 3.3. Experimental Results under Resting Conditions

**Figure 4:**
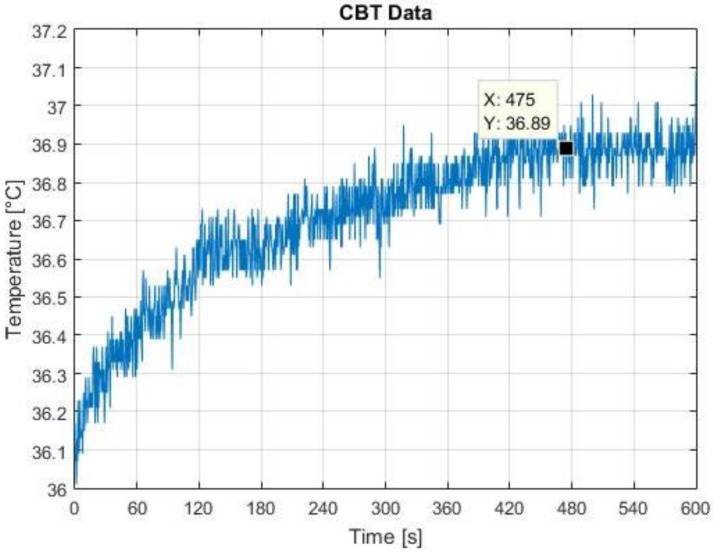
Raw temperature data acquired from the tympanic membrane.

Raw CBT data using the graphene-inked thermopile for one subject without any exercise are shown in Figure 4. The measured temperature rises gradually from 36 °C to an average of 36.89 °C after 8 min of data acquisition. The final average temperature approaches 37 °C which is within the normal CBT range of 36.5 °C to 37.5 °C for a healthy person. The raw temperature profile is similar for all subjects involved on this test. These data demonstrate the ability of the sensor to monitor CBT continuously for long periods of time.

The period of 8 min in which the measured temperature stabilizes is the time that the sensor takes to reach a thermal equilibrium. In steady state conditions, a precise measurement of the object temperature depends not only on the amount of IR radiation but also on the temperature of the sensor packaging which is measured and compensated for using an independent sensor. On the other hand, as soon as the IR thermopile is inserted into the ear, the surroundings of the ear canal begin to warm the sensor housing and therefore produces heat flow and thermal gradients in the sensor. According to Liess et al. [18], when an IR thermopile is under a non-thermal equilibrium, the sensor experiences a thermal shock that introduces a significant amount of errors to the measurements. Therefore, for continuous monitoring of CBT using an IR thermopile, it is necessary to wait for the sensor to achieve a thermal equilibrium, which enhances the accuracy of the measurements. 

The average temperature with the stabilized data was obtained for the ten subjects with both the original thermopile (Tth) and the graphene-inked thermopile (T_GN_) and analyzed against the Braun Thermoscan 7 IRTT (Tref). Lin’s Concordance Correlation Coefficient (CCC) obtained comparing Tth and Tref is rc = 0.150. Likewise, Lin’s CCC between the T_GN_ and Tref is rc = 0.163. Although both Lin’s CCC show a weak correspondence of the proposed device with and without graphene with regard to the reference thermometer, the graphene-inked thermopile shows slightly better concordance. This little improvement shows the better performance of the graphene-inked thermopile. However, to improve the outcomes of the graphene-inked thermopile, a more appropriate method for depositing the graphene coating over the lens of the sensor is suggested. 

**Figure 5:**
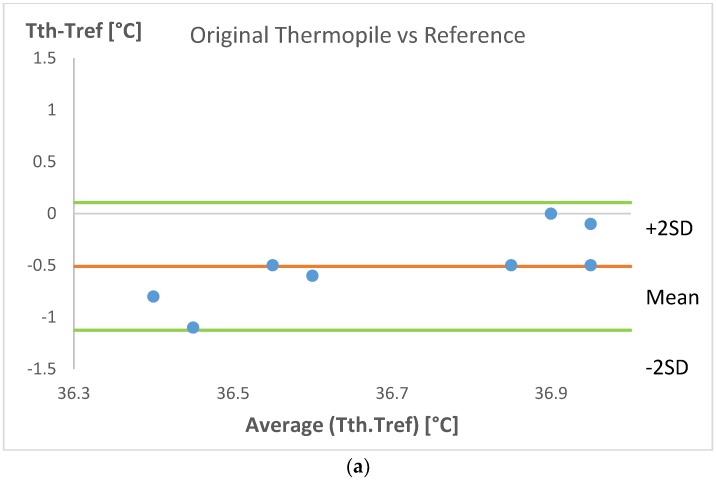

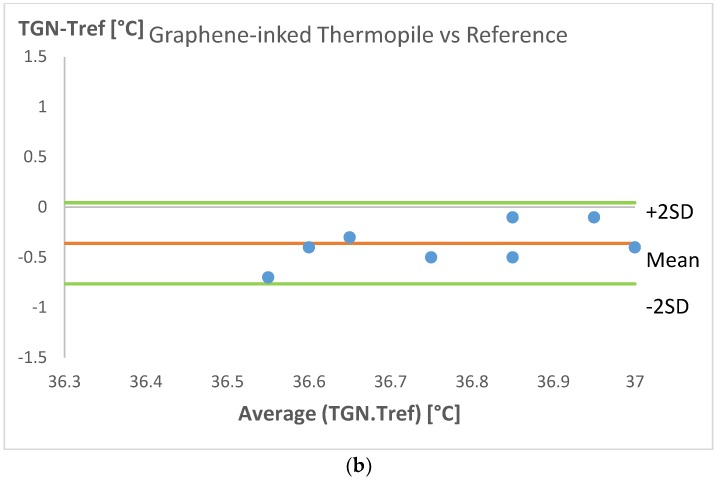
Bland–Altman plot between: (**a**) CBT acquired with the original thermopile and the reference thermometer; and (**b**) CBT measured with the graphene-inked thermopile and the reference thermometer.

Figure 5a,b shows the Bland–Altman analysis between the CBT gathered with Tref against CBT registered with Tth and T_GN_, respectively. The average temperature measured with the original thermopile was −0.51 °C below the Tref with ±0.62 °C 2 SD (Standard Deviation). On the other hand, the average temperature measured with the grapheme-inked thermopile was −0.36 °C below the Tref with ±0.40 °C 2 SD.

The reference Thermoscan 7 IRTT measured temperatures in the range from 36.80 °C to 37.30 °C with low SD around 0.17 °C. Results from the Bland–Altman analysis show that the original thermopile underestimates CBT by 0.51 °C, while the graphene-inked thermopile underestimates CBT by 0.36 °C. Therefore, the graphene-inked thermopile reduced the underestimation of CBT by 0.15 °C. The data from the Bland–Altman analysis show that the graphene coating could improve the transmission of IR radiation through the lens of the thermopile sensor.

The difference in measurements between the graphene-inked thermopile and the reference is due to the field of view (FOV) of the sensor and the anatomy of the ear. As the MLX90614-DCA sensor has a FOV of 90 °C [14], the thermopile not only detects radiation from the tympanic membrane but also from the auditory canal reducing the average CBT. Although there are thermopiles with smaller FOV, their larger dimensions do not allow an appropriate insertion into the ear canal. Another factor is the anatomy of the auditory canal. As the ear canal has an “S” shape that is different in every human, the sensor entered to a different depth in each participant. Therefore, lower temperature values are measured when the sensor is distant from the tympanic membrane.

### 3.4. Experimental Results under Physical Activity

**Figure 6:**
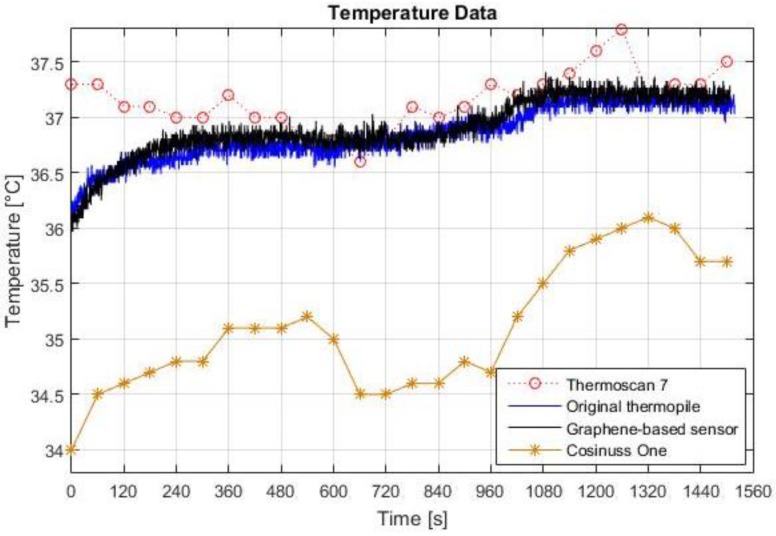
Temperature data during physical activity.

Figure 6 presents the measurements taken with the participant during 25 min. It shows the continuous readings acquired with the original and the graphene-inked thermopile as well as the readings taken each 60 s using the Thermoscan 7 IRTT and the Cosinuss One monitor. During the first 6 min, which represent the pre-heating stage, readings from all devices except for the reference thermometer present an increasing behaviour up to a stable value. The following 10 min represent the exercising period. During this stage, an initial decrease of the measured temperature is observed with the four devices. Finally, after the exercising stage, body temperature increases to a value higher than the temperature before the physical training stage. 

Results in Figure 6 show an apparent enhancement in the performance of the proposed device due to the use of the GN-inked thermopile. It can be appreciated that the measured temperature profile using the GN-based thermopile is slightly higher than the profile acquired using the original MLX90614-DCA thermopile. The calculated average of the differences between both thermopiles shows that the graphene-inked thermopile measures a temperature 0.06 °C higher than the temperature acquired with the original thermopile.

The core body temperature measured with the graphene-inked sensor has an expected behaviour. There is an initial decrease from 36.8 °C to 36.6 °C during the first four minutes of physical activity. This pattern is due to the triggering of thermoregulation responses of the body. At the beginning of the exercise period, blood flow increases to the skin and skeletal muscles and as a result heat begins to be dissipated causing a small reduction of core temperature [19]. Nevertheless, as the subject keeps performing physical activity, the heat produced is accumulated in the body and the CBT increases as expected. It can be observed that, after 8 min of exercise, CBT rises to a final value of around 37.3 °C. All things considered, the graphene-inked sensor is capable of detecting changes in CBT.

This test demonstrates the better performance of the graphene-inked sensor against the Cosinuss One commercial body monitor. The Cosinuss One presents a temperature measurement 1.5 °C lower than the reference CBT through the entire test. In contrast to the Cosinuss One monitor, the graphene-inked sensor is less likely to be influenced by airflow and ambient temperature. 

## 4. Conclusions

In this study, an in-ear wearable device that measures CBT from the tympanic membrane was designed and constructed based on a graphene-coated IR thermopile sensor, and its performance was tested against two commercial ear thermometers. The earbud as well as the device enclosure were designed using CAD software and manufactured through STL 3D printing technology. According to the experimental results, the proposed GN-coated IR thermopile sensor measured CBT from inner ear with increased accuracy compared commercial IR thermopiles. Furthermore, a smartphone app was designed to receive CBT from the proposed device wirelessly to display and plot the obtained data. The developed device is able to monitor CBT for long periods of continuous use even during exercise periods.
